# The role of atypical MAP kinase 4 in the host interaction with *Cryptosporidium parvum*

**DOI:** 10.1038/s41598-023-28269-w

**Published:** 2023-01-19

**Authors:** Nina Watanabe, Hironori Bando, Fumi Murakoshi, Riku Sakurai, Mohammad Hazzaz Bin Kabir, Yasuhiro Fukuda, Kentaro Kato

**Affiliations:** 1 Laboratory of Sustainable Animal Environment, Graduate School of Agricultural Science, Tohoku University, 232-3 Yomogida, Naruko-Onsen, Osaki, Miyagi 989-6711 Japan; 2 Department of Parasitology, Asahikawa Medical University, 2-1-1-1, Midorigaoka-Higashi, Asahikawa, Hokkaido 078-8510 Japan; 3 Department of Infectious Diseases, Graduate School of Medical Science, Kyoto Prefectural University of Medicine, 465, Kawaramachi-Hirokoji, Kamigyo-ku, Kyoto, 602-8566 Japan

**Keywords:** Microbiology, Parasitology

## Abstract

*Cryptosporidium parvum* is an apicomplexan parasite that causes severe zoonotic diarrhea in humans and calves. Since there are no effective treatments or vaccines for infants or immunocompromised patients, it is important to understand the molecular mechanisms of the parasite–host interaction for novel drug discovery. Mitogen-activated protein kinase (MAP kinase) is a key host factor in interactions between host and various pathogens, including parasites. Although the function of conventional MAP kinases against parasite infection has been investigated, that of atypical MAP kinases remains largely unknown. Therefore, we focused on one of the atypical MAP kinases, MAPK4, and its effect on *C. parvum* infection in human intestinal cells. Here, we report that MAPK4-deficient intestinal cells showed a significant reduction in *C. parvum* infection. We also show that host MAPK4 has a role in host cell survival from *C. parvum* infection. In addition, we show that *C. parvum* requires host MAPK4 for its successful invasion and asexual reproduction. Taken together, our data suggest that MAPK4 is an important host factor contributing to *C. parvum* infection in human intestinal cells.

## Introduction

*Cryptosporidium parvum* is a protozoan parasite that infects intestinal epithelial cells and causes zoonotic waterborne diarrhea, cryptosporidiosis^1^. Cryptosporidiosis has a substantial impact on livestock production, and is a constant human health concern in both economically developed and developing countries^2–4^. It is especially related to the global morbidity and mortality of infants exposed to lower quality water sanitation, as the fifth leading diarrheal pathogen for children under 5 years old in 2016^5^. Despite these concerns, only one drug, nitazoxanide, is approved by the U.S. FDA for the treatment of cryptosporidiosis, suggesting that more basic research on *C. parvum* may be needed^6,7^. Especially, an in-depth understanding of its biology and the parasite–host relationship are urgently required for the development of novel cryptosporidiosis treatments.

*Cryptosporidium parvum* enters the small intestine as a hard-shelled oocyst and releases four motile sporozoites to invade the intestinal host cells^8^. Upon successful invasion, the parasite causes membrane protrusions enabling its encapsulation and formation of intracellular but extracytoplasmic distinct structures called parasitophorous vacuoles (PVs). Subsequently, invaded sporozoite transforms into a trophozoite, a round intracellular growth stage, and multiplies asexually into a meront, a reproductive stage with two to eight nuclei, which releases eight motile merozoites from the infected cell^9^. Previous studies have shown that various host factors are involved in each stage of infection. For example, parasite invasion requires a change in the structure of the host cell membrane via cdc42 GTPase signaling and the intestinal N-WASP network^10,11^. Parasites also alter host mechanisms of glucose transport to ensure successful nutrition uptake^12^. In addition, a long noncoding RNA NR_033736 is recruited to downregulate anti-*Cryptosporidium* innate immunity in host enterocytes, contributing to successful parasite development^13^. Despite the various findings, little is known about the overall molecular mechanisms involved in the interaction between *C. parvum* and its host cells.

The mitogen-activated protein kinase (MAP kinase) family consists of highly conserved protein kinases in eukaryotes that are known to play a central role in intercellular signal transduction^14^. The family includes 14 kinases with seven groups, classifying four atypical members separately from ten conventional members due to nonconforming particularities^15^. Conventional MAP kinases such as ERK1/2, JNK1/2/3, and the p38 isoforms have been extensively studied and characterized as essential factors for innate immunity and infectious strategies of various pathogens^16,17^. For example, p38 MAPK ensures resistance of *Caenorhabditis elegan*s to bacterial infection, by regulating the stress-responsive cascade^18^. The JNK pathway in murine macrophages also mediates TLR2-dependant ROS signaling as an inflammatory response to Mycobacterium infection^19^. Conversely, PAK-MAPKK-ERK1/2 signaling in host cells is required for the survival of *Plasmodium* parasites, in two distinct stages in human erythrocytic cells and mice liver cells^20^. Conventional MAP kinases are also involved in the *C. parvum*-host interaction. Under *C. parvum*-mediated stimuli, ERK1/2 and p38 MAPK signaling in bovine and human polymorphonuclear cells (PMCs) is required for the host immune response of neutrophil extracellular trap (NET) formation^21^. Conversely, *C. parvum* infection suppresses the expression of host p38 MAPK in murine intestinal epithelial cells, while in presence of pan-MAPK activator anisomycin, parasite burden at 24 h post-infection is reduced despite the higher rates of attachment and invasion^22^. Taken together, these results demonstrate that MAP kinases play a diverse and important role in pathogen-infected host cells.

In contrast to conventional MAP kinases, the biological significance of atypical MAP kinases in the parasite–host interaction has been overlooked. A transcriptome survey reported that an atypical MAP kinase called MAPK4 (also known as ERK4) shows lower expression in popliteal lymph node aspirates of dogs infected with the protozoan parasite *Leishmania infantum*^23^; however, the contribution of MAPK4 in infection has never been investigated. Therefore, in this study, we focus on host MAPK4 to reveal its involvement in protozoan parasite–host interactions by performing phenotypic analyses of MAPK4-deficient human intestinal cells.

In the present study, we demonstrated that host MAPK4 is involved in the pathogen-host interaction in human cells. The infection rate of *C. parvum* was significantly reduced in MAPK4-deficient host cells, which coincided with an increase in parasite-induced apoptosis, and a reduction in parasite invasion and asexual reproduction. Taken together, our study reveals that host MAPK4 has diverse and important roles in the *C. parvum*-host interaction in human intestinal epithelial cells.

## Results

### Establishment of a MAPK4-deficient HCT-8 cell line

**Figure 1:**
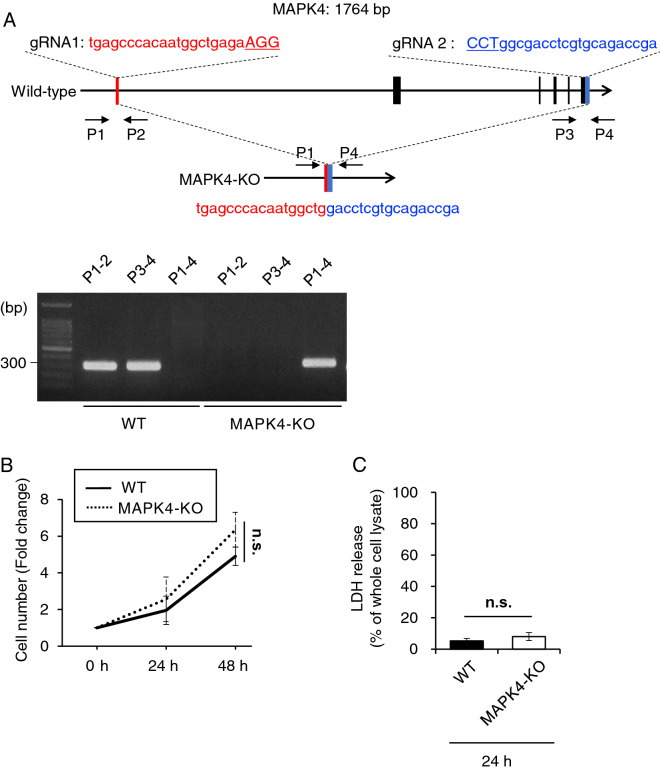
Generation of MAPK4-KO HCT-8 cells. (**A**) Schematic image of the strategy to generate MAPK4-KO HCT-8 cells and genotyping. Two gRNAs were designed to hybridize and eliminate the MAPK4-coding site in HCT-8 cells. Genomic DNA of each cell was extracted and analyzed by PCR analysis. PCR of P1-4 site was not amplified with WT HCT-8 cells because of long introns in-between. The sequence of the P1-4 product in MAPK4-KO HCT-8 cells is shown in the image. (**B**) Cell growth assay of WT and MAPK4-KO HCT-8 cells. No significant difference was observed with cell types (Two-way ANOVA, *p* = 0.1316). (**C**) LDH-based cell mortality of WT and MAPK4-KO HCT-8 cells under non-infected conditions. Percentages represent the ratio of LDH release to whole cell lysates. No significant difference was observed (Student’s t test, *p* = 0.1393). All graphs show the mean ± SEM for more than three independent experiments. All images are representative of three independent experiments.

To examine the role of host MAPK4 on parasite infection, we generated MAPK4-deficient (MAPK4-KO) human intestinal adenocarcinoma HCT-8 cells by using CRISPR/Cas9-genome editing. Two pairs of guide RNAs were designed and expressed with the Cas9 protein in HCT-8 cells. Genomic PCR of the subcloned cell line showed amplification of P1-4 sites, which is not able to be amplified in WT HCT-8 cells because of interruption of introns longer than the maximum limit of PCR analysis (for example, the first two exons are separated by a long intron of 102.8 kb). Sequence analysis confirmed that 1715 bp of the 1764 bp MAPK4 coding region including some introns was eliminated between the designed gRNA pairs in the genome of MAPK4-KO HCT-8 cells. Therefore, we concluded that the MAPK4-KO HCT-8 cell line was successfully established (Fig. 1A).

Previous studies have shown that host MAPK4 deficiency leads to suppression of cell proliferation or an increase in apoptosis in several cell types^24^. To test whether MAPK4 deficiency effected the viability of the HCT-8 cell line, we performed an MTT-based assay and a lactate dehydrogenase (LDH)-based assay. No significant difference was observed between wild-type (WT) and MAPK4-KO HCT-8 cells in terms of both cell proliferation and cell death (Fig. 1B,C), suggesting that MAPK4 does not affect HCT-8 cell viability under non-infected conditions.

### MAPK4 deficiency significantly reduces *C. parvum* infection

**Figure 2:**
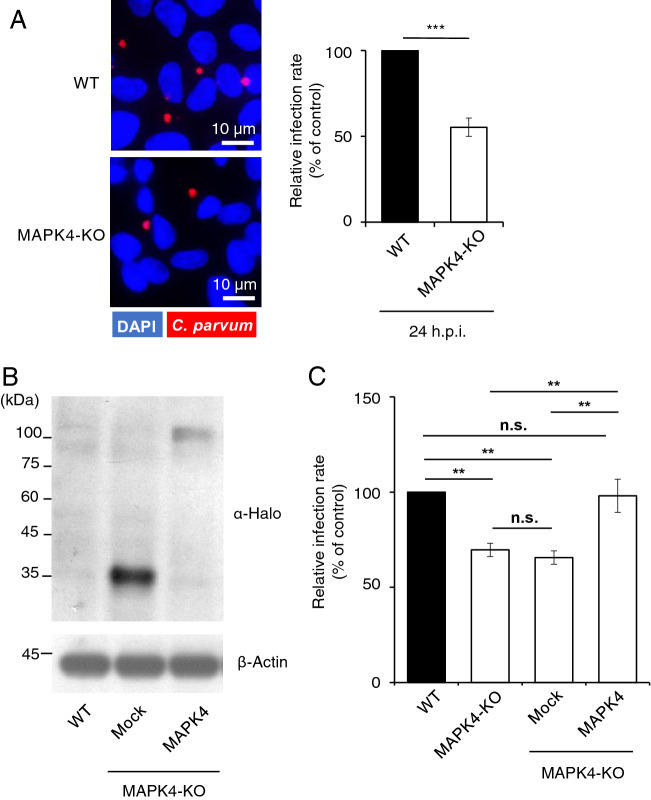
Reduction of the infection rate in *C. parvum-*infected MAPK4-KO HCT-8 cells. (**A**) Effect of host MAPK4 deficiency on *C. parvum* infection in HCT-8 cells. The infection rates were analyzed by use of an immunofluorescence assay at 24 h post-infection. The number of *C. parvum-*infected (red; Sporo-Glo) and total host cells (blue; DAPI) was counted by microscopy, and the infection rates are shown as the ratio of *C. parvum*-infected amongst total host cells and normalized to the WT HCT-8 cells control**.** ***p < 0.001 (Student’s t test). (**B**) Transient expression of MAPK4 protein in MAPK4-KO HCT-8 cells. Protein extracts from WT and transfected MAPK4-KO HCT-8 cells were analyzed by western blotting. HaloTag protein (33 kDa) and MAPK4-HaloTag protein (101 kDa) were detected with an anti-HaloTag antibody. (C) Effect of transient expression of MAPK4. MAPK4-KO cells were transfected with empty HaloTag vector or the MAPK4 expression vector, and infected with *C. parvum* oocysts. The experiment was conducted in 96-well plates. The infection rates were calculated as a proportion of infected cells among total host cells. **p < 0.01, *p* = 0.9005 (WT—KO + MAPK4), *p* = 0.9892 (KO—KO + Mock) (Tukey–Kramer method). All graphs show the mean ± SEM for three independent experiments. All images are representative of three independent experiments.

To investigate whether host MAPK4 is required for *C. parvum* infection, we compared infection rates between WT and MAPK4-KO HCT-8 cells at 24 h post-infection as a representative of the parasite’s asexual cell cycles^9^. The infection rate was calculated as a proportion of infected cells among total host cells, and was detected using a polyclonal antibody against all developmental stages^25^. The infection rate decreased by 45% as a result of MAPK4 knock out (Fig. 2A). The observed reduction in infection rate suggests that host MAPK4 is an important host factor utilized by *C. parvum* to establish infection. QRT-PCR analysis of *C. parvum* infection was also performed to ensure the phenomena, demonstrating the similar result of almost half the infection rates of MAPK4-KO cells over those of WT HCT-8 cells (Fig. S2). To further confirm the significance of MAPK4 expression in host cells, we rescued MAPK4 expression in MAPK4-KO HCT-8 cells by transient transfection using a MAPK4-HaloTag expression vector. Expression of the HaloTag fusion MAPK4 protein in the transfected MAPK4-KO HCT-8 cells was confirmed by western blotting with an anti-HaloTag antibody (Fig. 2B). The exogenous overexpression of MAPK4 rescued the infection rate to 95% of that of WT cells, compared with mock-transfected MAPK4-KO HCT-8 cells at 65% (Fig. 2C). The reduction of parasite infection in MAPK4-KO cells was assessed to be minor due to the smaller scale using 96-well plates, but the infection rate was significantly reduced in MAPK4-KO and Mock-transfected MAPK4-KO cells, compared to both WT HCT-8 cells and MAPK4-KO HCT-8 cells transiently overexpressing MAPK4. Taken together, these data confirm the importance of host MAPK4 in *C. parvum* ability to infect intestinal cells.

### Host MAPK4 is involved in the regulation of ***C. parvum***-induced cell death in HCT-8 cells

**Figure 3:**
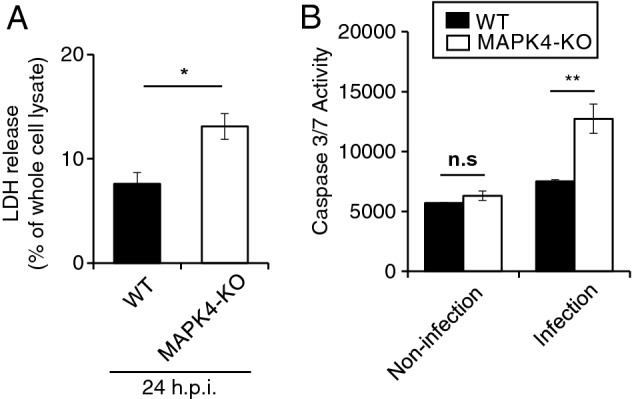
Increased host cell death and caspase 3/7 mediated-apoptotic cell death in *C. parvum-*infected MAPK4-KO HCT-8 cells. (**A**) LDH-based mortality of *C. parvum*-infected WT and MAPK4-KO HCT-8 cells at 24 h post-infection. Percentages represent the ratio of LDH release to whole cell lysates. *p < 0.05 (Student’s t test). (**B**) Apo-ONE homogeneous caspase 3/7 activity in *C. parvum*-infected WT and MAPK4-KO HCT-8 cells measured 24 h post-infection. No significant difference was observed between non-infected cells (Student’s t test, *p* = 0.1412). *p < 0.05 (Student’s t test). All graphs show the mean ± SEM for three independent experiments.

*Cryptosporidium parvum* modulates host immune responses, including apoptosis, to facilitate its survival and propagation^26^. MAPK4 signaling has a significant role in the regulation of apoptosis in various human cells^24,27^. Therefore, we first examined the effect of host MAPK4 on *C. parvum*-induced cell death by using LDH cytotoxicity assay. There was no change in viability of MAPK4-KO HCT-8 cells in the absence of *C. parvum* (Fig. 1C), whereas infected MAPK4-KO HCT-8 cells showed a 2.3-fold higher rate of cell death compared with infected WT HCT-8 cells at 24 h post-infection (Fig. 3A). These results suggest that host MAPK4 is involved in the signal pathway of *C. parvum*-induced cell death in human intestinal epithelial cells.

The leakage of intracellular LDH is an indicator of plasma membrane breakdown, which is a consequence of various types of cell death^28,29^. Caspase-dependent apoptosis, terminated with enzymes called caspase 3 and 7, is known to be required for apoptosis progression and modulation in *C. parvum*-infected cells^30,31^. To examine whether caspase-dependent cell death is induced or inhibited in infected MAPK4-KO HCT-8 cells, we next analyzed the activity of caspase 3/7. As a result, both WT and MAPK4-KO HCT-8 cells showed higher caspase 3/7 activity under *C. parvum* infection (*p* = 0.0003 and *p* = 0.0074 each), consist with previous research^30,32^. However, the propensity was more enhanced in MAPK4-KO HCT-8 cells showing 2.0 times higher caspase 3/7 activity than that of infected WT HCT-8 cells at 24 h post-infection, whereas no significant difference was observed under non-infected conditions (Fig. 3B).

These data indicate an involvement of host MAPK4 in the regulation of caspase-dependent apoptotic cell death in *C. parvum-*infected intestinal human cells.

### Host MAPK4 is involved in parasite invasion and asexual reproduction, but not attachment in HCT-8 cells

The MAP kinase family is involved in diverse signaling pathways, including stress responses, membrane integrity, cytoskeletal reorganization, protein localization, and metabolism^15,16,33–36^. Therefore, to examine whether MAPK4 is involved in the life cycle progression of *C. parvum*, we performed a comparison analysis of the parasite phenotypes of attachment, invasion, and asexual reproduction stages in the presence or absence of host MAPK4.

**Figure 4:**
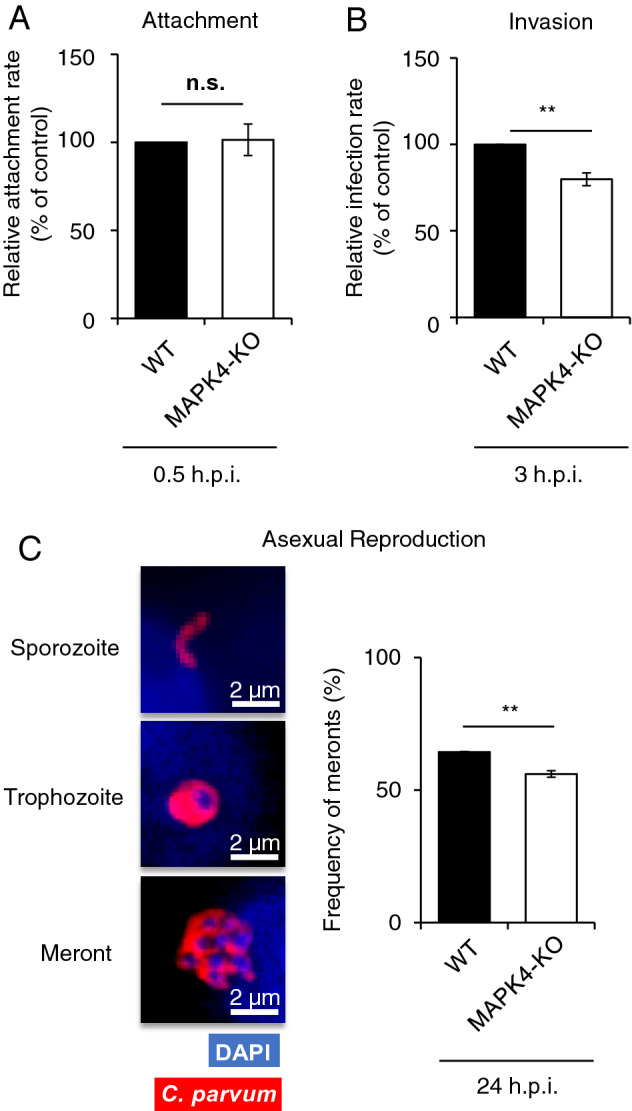
Inhibitory effects on the invasion and asexual reproduction stages of *C. parvum* in infected MAPK4-KO HCT-8 cells. (**A**) Effect of host MAPK4 deficiency during the attachment. Pre-fixed HCT-8 cells were inoculated with *C. parvum* sporozoites. The attachment rates were analyzed by use of an immunofluorescence assay at 30 min post-infection. The attachment rates are shown as the ratio of *C. parvum*-attached host cells amongst total host cells and normalized to the WT HCT-8 cells control**.** No significant difference was observed (Student’s t test, p = 0.4495). (**B**) Effect of host MAPK4 deficiency on invasion. The infection rate was analyzed 3 h post-infection by means of immunofluorescence assay. The invasion rates are shown as the ratio of *C. parvum*-infected host cells amongst total host cells and normalized to the WT HCT-8 cells control**.** *p < 0.05 (Student’s t test). (**C**) Effect of host MAPK4 deficiency on asexual reproduction. Parasite stages were identified microscopically by parasite staining (red; Sporo-Glo) and nuclear staining (blue; DAPI) as either a trophozoite (with a single nucleus) or a meront (with 2–8 nuclei). The ratio of meronts to total counted parasites is shown as the frequency of meronts. At least 50 parasites were counted per coverslip. Banana-shaped sporozoites were not counted as infected parasites in this assay. **p < 0.01 (Student’s t test). All graphs show the mean ± SEM for three independent experiments.

The *C. parvum* sporozoite adheres to the surface of intestinal epithelial cells via specific host receptors and its membrane structure^37–39^. First, we assessed whether host MAPK4 deficiency influences the very first step of parasite infection; attachment. The attachment rate of sporozoites was calculated by means of immunofluorescence assay, as the proportion of attached parasite per total of fixed host cells. WT and MAPK4-KO HCT-8 cells showed no significant difference in the sporozoite attachment rate (Fig. 4A), suggesting that host MAPK4 does not contribute to the attachment of *C. parvum* sporozoites to the host plasma membrane. The viability of processed sporozoites were analyzed by immunofluorescence assay with un-fixed WT and MAPK4-KO HCT-8 cells, ensuring the parasite development in host cells and the reduction of infection rate in MAPK4-KO cells after processed sporozoite infection (Fig. S3).

MAP kinases have a prominent role in both cytoskeleton dynamics and protein translocation^40^. After the attachment of sporozoites, parasites require the accumulation of host cytoskeletal proteins and glucose transporters to create a dynamic protrusion from the host plasma membrane^41,42^. To assess whether MAPK4 has a role in parasite invasion, we examined the effect of host MAPK4 deficiency on the invasion stage of *C. parvum* at 3 h post-infection. The infection rate was significantly decreased in MAPK4-KO HCT-8 cells by 20% (Fig. 4B), suggesting that host MAPK4 has an effect on the invasion of *C. parvum*.

Within 36 h of *C. parvum* sporozoite invasion, motile merozoites are released as a result of asexual reproduction, called merogony^8^. Accordingly, we investigated the effect of host MAPK4 on the reproduction of *C. parvum* at 24 h post-infection, by counting the number of *C. parvum* in each phase of merogony in host cells. Relative proportions of meronts, showing a larger globular shape with multiple nuclei, to developing trophozoites, showing a globular shape with a single nucleus, were determined. The reproduction rates were 64.37% ± 0.1860 in WT HCT-8 cells and 56.06% ± 1.240 in MAPK4-KO HCT-8 cells, indicating an 8% reduction in the reproduction rate caused by host MAPK4 deficiency (Fig. 4C) and suggesting that host MAPK4 influences the asexual reproduction of *C. parvum*. Taken together, our data demonstrate that host MAPK4 has diverse roles in *C. parvum* infection of human intestinal epithelial cells.

## Discussion

In the present study, we confirmed the involvement of host MAPK4 in pathogen-host interactions in human intestinal cells. Absence of MAPK4 in host cells led to a significant reduction in the *C. parvum* infection. We demonstrate this is related to promotion of infection-induced host cell death, reduction in invasion of sporozoites, and reduction in asexual reproduction of intercellular parasite stages. These results provide the first evidence of a parasite utilizing the host MAPK4 to establish infection.

Although we revealed that the function of MAPK4 relates to *C. parvum*-host interaction, the functional basis for this interaction remains unclear. MAPK4 is characterized by a unique phospho-receptor motif (S-E-G instead of T-x-Y) and is speculated to have different switches and substrates than conventional MAP kinases. Therefore, there is a possibility that the kinase activity of MAPK4 is related to role in the *C. parvum*-host interaction^24,43^. Indeed, a recent study with various human cells revealed that MAPK4 activates a downstream cascade via the noncanonical activation of AKT/mTOR signaling^24^. Thus, host MAPK4 may directly support *C. parvum* by signaling with downstream factors. Conversely, back-spliced circular RNA MAPK4 inhibits conventional MAPK signaling by sponging miR-125a-3p in human multiple myeloma MM.1S and H929 cells^27^. Thus, the transcriptional product from the host MAPK4 coding site may affects the parasite indirectly by regulating the expression of other MAP kinases in MAPK4-KO cells. Future studies are needed to elucidate whether the reduction of *C. parvum* infection in MAPK4-KO HCT-8 cells is due to the kinase activity, an RNA-based regulatory function, or a combination of both, to understand both the function of host MAPK4 signaling and the parasitic strategy of *C. parvum* that allows it to benefit from that function.

It is well-known that cell death is an important host immune response for pathogen control^44^. Caspase-dependent apoptotic cell death of the host has been reported as one of the regulatory factors of *C. parvum* infection^45^. In addition, it has been suggested that *C. parvum* modulates host apoptosis; however molecular mechanisms of this relationship remain unknown^26,30^. In this study, we once confirmed that *C. parvum* infection induces caspase-dependent apoptotic cell death in human intestinal epithelial cells as previously revealed, and further demonstrated that host MAPK4 is involved in its modulation. Various anti-apoptotic mechanisms have been reported, and several molecules have been identified as *C. parvum*-regulated host factors. For example, Bcl-2, an inhibitor of pro-apoptotic protein Bax, and survivin (also known as BIRC5), a member of the Inhibition of Apoptosis Protein (IAP) family, are known to play a key role in regulating *C. parvum*-induced apoptosis^32,46^. Both genes are sensitive to eIF4E-mediated translational regulation, which is mainly regulated by AKT/mTOR signaling, which is directly regulated by MAPK4^47^. Furthermore, MAPK4, Bcl-2, and survivin are among the 39 genes that were upregulated in a human rectal cancer patient trial^48^. Thus, our data may suggest that host MAPK4 is involved in anti-apoptotic signaling during *C. parvum* infection.

After invasion into host intestinal cells, *C. parvum* undergoes asexual cycle within 36 h. Sporozoite invasion is preceded by its adherence to the host cell and remodeling of its membrane. Invasion and encapsulation in PV results in formation of an intracellular trophozoite, which morphs into a multinucleated meront by means of asexual replication^1^. We investigated the effect of MAPK4 on each of these stages of infection. Our findings showed that invasion and asexual reproduction, but not attachment of *C. parvum* are affected in the absence of host MAPK4. Adhesion of sporozoites to the host cell surface is the first step of infection. Since the parasite attachment is not affected by host MAPK4 deficiency, it is hypothesized that the effect of host MAPK4 on parasite invasion is resulted by a subsequent biological process after attachment. Predicted functions of MAPK4 include regulation of the cytoskeleton, which is considerably important for the PV formation of *C. parvum*^15,49^. For example, when a motile sporozoite attaches to the apical membrane of the host epithelial cell, host actin and its terminal web, LMO7, are recruited to create the host cell membrane protrusion^41,50^. Therefore, disorder at the cytoskeleton organization level due to MAPK4 deficiency may have contributed to the reduced invasion rate. On the other hand, limited information is available to speculate the reason for the developmental inhibition of the parasites in MAPK4-KO HCT-8 cells. Conventional MAP kinases are known to be involved in metabolic regulation^51^. *C. parvum* acquires necessary nutrients from host cells and the gut environment while affecting the host metabolic pathways^52–54^. Thus, the variation in metabolic changes in MAPK4-KO HCT-8 cells could be the reason for the inhibition of the asexual reproduction of the parasites. Additionally, there is some evidence that conventional MAP kinases are involved in intracellular trafficking, suggesting the possibility of transport defects due to MAPK4 deficiency^33,40^. Our interest is the possibility of incomplete formation of feeder organelles, which are dense band-like structures between the parasites and host cells used for transporting and exporting nutrients^55^. Although the formation of the protozoan microstructure was not examined in this study, it is possible that the delayed asexual reproduction is a result of the unsuccessful invasion, coupled with inefficient construction of microstructure within host cells. Further studies are demanded to fully understand the effects of host MAPK4 signaling on *C. parvum* invasion and development in host intestinal cells.

Although this study has revealed the importance of MAPK4 in parasite–host interactions in *C. parvum*-infected cells, the mechanism of parasite interference with host MAPK4 remains to be elucidated. Although the specific pathway for MAPK4 activation is not clearly understood, two possible strategies of parasites are considered. First is ligand-receptor interactions during invasion. MAPK4 is known to be activated by p21-activated kinase (PAK), which is located downstream of Rho family small GTP-binding proteins (GTPases)^56^. Cdc42 is a kind of small GTPases acting as a major regulator of cytoskeletal reorganization during *C. parvum* invasion^57^. Therefore, host MAPK4 may be activated by the same mechanism by which Cdc42 is activated and plays an important role through Cdc42-related signaling. The other possible strategy is virulence factors delivered into the host cytoplasm from parasites. Recently, it has been suggested that *C. parvum* injects proteins and non-coding RNAs to manipulate its host^50,58^. Since MAPK4 is the terminal enzyme of the MAP kinase cascade and is thought to be intricately regulated through transcription, translation, and activation, there may be agents from *C. parvum* that interfere with these mechanisms^24,59^. Future investigation for those ligands or agents involved in the possible interference by parasites for manipulating host MAPK4 will facilitate potential drug targets for inhibiting parasitic infection and growth in infected mammals.

In summary, this study is the first to report an infectious event in which host MAPK4 has an important role. We revealed diverse effects of host MAPK4 on parasite infection; however, underlaying mechanisms of MAPK4 signaling are yet to be investigated. Future analyses of the function and activation of host MAPK4 in *C. parvum*-infected cells might help us elucidate a novel mechanism of the virulence of *C. parvum* and unknown functions of MAPK4. This research has potential to promote future development of novel anti-cryptosporidium therapies that inhibit invasion and growth of this protozoan parasite, along with the insight of novel function of an atypical MAP kinase.

## Materials and methods

### Cells and parasites

Human ileocecal colorectal adenocarcinoma (HCT-8) cells (American Type Culture Collection (ATCC), Manassas, VA, USA) were maintained in RPMI-1640 medium (Nacalai Tesque, Kyoto, Japan) supplemented with 10% heat-inactivated fetal bovine serum (JRH Biosciences, Lenexa, KS, USA), 100 U/mL penicillin, and 0.1 mg/mL streptomycin (Nacalai Tesque). All cultures were maintained at 37 °C under 5% CO_2_.

*Cryptosporidium parvum* oocysts, strain HNJ-1, were kindly provided by Dr. M. Matsubayashi (Osaka Prefecture University). Oocysts were maintained by passage in severe combined immunodeficiency (SCID) mice (Jackson Laboratory Japan, Yokohama, Japan) and purified from feces by sucrose suspensions and cesium chloride centrifugation as previously described^60^.

### Establishment of the MAPK4-deficient HCT-8 cell line

The MAPK4-deficient (MAPK4-KO) HCT-8 cell line was generated with the px330 CRISPR/cas9 system, as previously described^61^. Briefly, two insert fragments of guide RNAs (gRNAs) were generated by annealing the primers (gRNA1_F and gRNA1_R primers, and gRNA2_F and gRNA2_R primers, respectively, listed in Table 1) and inserting them into the *Bbs*I sites of cloning vectors containing the U6 promoter, thereby generating gRNA expressing plasmids (pMAPK4_gRNAn). An insert fragment cut out by *Xho*I and *Sal*I from pMAPK4_gRNA2 was ligated into the *Xho*I site of pMAPK4_gRNA1 to generate pMAPK4_gRNA1/2, and an insert fragment cut out by *Kpn*I and *Mlu*I from the completed gRNA plasmids was ligated into the pEF6-hCas9-Puro vector to create an all-in-one pMAPK4_ gRNA1/2_Cas9 vector. The sequence was confirmed by sequencing analyses using an ABI PRISM Genetic Analyzer 3130xl (PE Applied Biosystems). The primers used for plasmid construction and sequencing are listed in Table 1. The molecularly confirmed plasmid was transfected into HCT-8 cells using Lipofectamine^®^ 3000 reagent (Invitrogen, Waltham, MA, USA) and incubated for 12 h, followed by at least 3 days of antibiotic selection under 10 µg/ml puromycin (Nacalai Tesque). Cells were subcloned by limiting dilution in 96-well plates, and a MAPK4-KO single-cell derived clone was isolated.

**Table 1:** Primer list.

Primer	Sequence (5′–3′)
GRNA1_F	CACCGTGAGCCCACAATGGCTGAGA
GRNA1_R	CACTCGGGTGTTACCGACTCTCAAA
GRNA2_F	CACCGTCGGTCTGCACGAGGTCGCC
GRNA2_R	CAGCCAGACGTGCTCCAGCGGCAAA
P1	ACTAGGAGAAAACACATCCCTCA
P2	ATCGCTCAGGGCAATCTTCTTCA
P3	CAGTTCGACCTGGACGTGTTCAT
P4	AATGGACATTCCTCTCGCTCCTC
COMP_F	GGATCCGCCACCATGGCTGAGAAGGGTGACTGCAT
COMP_R	GCGGCCGCCCACCTTTCTTTGGAGAAGGCCTCGGT
CP18S-1011F	TTGTTCCTTACTCCTTCAGCAC
CP18S-1011F	TCCTTCCTATGTCTGGACCTG
HS18S-1F	GGCGCCCCCTCGATGCTCTTA
HS18S-1F	CCCCCGGCCGTCCCTCTTA

### Genomic PCR and sequencing analyses

The obtained monoclonal cell line was identified as MAPK4-KO cells by genomic PCR and sequencing analyses using the ABI PRISM Genetic Analyzer 3130xl (PE Applied Biosystems, Waltham, MA, USA). Primer pair P1 and P4 (P1–4) region (amino acid #138 at the promoter site to amino acid #1914 after the terminal codon), which can only be amplified with MAPK4-KO cells because long introns in human genome (for example, the first two exons are separated by a long intron of 102.8 kb) would interrupt PCR while all the introns is totally eliminated by gene editing in MAPK4-KO HCT-8 cells, was analyzed by 1.5% agarose gel electrophoresis in TAE buffer. The absence of human MAPK4 coding site in MAPK4-KO HCT-8 cells was confirmed by PCR of the primer pair P1 and P2 (P1-2) for the coding sequence of the N-terminal (308 bp, amino acid #138 at the promoter site to amino acid #165) and P3 and P4 (P3-4) for the C-terminal (309 bp, amino acid #1605 to amino acid #1914 after the terminal codon) regions before each experiment, with the primer pair P1 and P4 (P1–4) region as a positive control, by using KOD FX Neo PCR and KOD One enzymes (Toyobo, Osaka, Japan) according to the manufacturer's instructions. The primers used are listed in Table 1.

### Cell viability assay

The cell number and the ratio of cell death were measured using Cell Count Reagent SF (Nacalai Tesque) and the CytoTox 96^®^ Non-Radioactive Cytotoxicity Assay system (Promega Corporation, Madison, WI, USA) according to the manufacturer's instructions. Briefly, WT and MAPK4-KO HCT-8 cells were seeded in 96-well plates (5 × 10^4^ cells/well). Then, cell proliferation was assessed by evaluating total cell numbers at 0, 24, and 48 h after seeding. Cell mortality under non-infected conditions was measured by LDH activity in the cell culture supernatant at 24 h after seeding. Cell mortality under infected conditions was also measured by LDH activity of the cell culture supernatant at 24 h post-infection. The absorbance was measured by using a Multi Detection Microplate Reader (Power scan HT; DS Pharma Biomedical, Osaka, Japan). Cell-free medium was used as a background control, while cell culture supernatant from the cells treated with TritonX-100 (0.1%) was used as a positive control. The LDH release for each sample was normalized to the positive control as 100% dead.

### Preparation of purified oocysts and excysted sporozoites

*Cryptosporidium parvum* oocysts were bleached with 10% (v/v) purelox (OyaloxCo.Ltd., Tokyo, Japan) on ice for 15 min, then washed 3 times with ice-cold phosphate buffered saline (PBS) and incubated with 0.2 mM sodium taurocholate (Nacalai Tesque) at 15 °C for 10 min to stimulate excystation. For the attachment assay, oocysts were further incubated at 37 °C for 1 h and filtered through a 5-μm pore-size PVDF filter (Millipore, Burlington, MA, USA) to separate excysted sporozoites. Pre-excysted oocysts or purified sporozoites were counted under a microscope using a hemocytometer. Each culture group was infected with the oocysts or sporozoites at the same rate of infection in each experiment (i.e., at a multiplicity of infection of 0.5–2).

### Invasion and infection assay

Coverslips were coated with 100 µg/ml poly-l-lysine (Nacalai Tesque) for 30 min at 37 °C, washed 3 times with sterilized water, and dried under UV light for at least 15 min. HCT-8 cells were seeded on coverslips (4 × 10^5^ cells/well) or 96-well plates (5 × 10^4^ cells/well) and incubated overnight before infection*. C. parvum* infection was performed as described above. Infected cultures were incubated at 37 °C under 5% CO_2_ for 3 h for the invasion assay and 24 h for the infection assay. After the incubation, each culture was washed 3 times with ice-cold PBS, fixed in 3.7% formaldehyde in PBS at room temperature for 15 or 30 min, permeabilized with ice-cold 100% methanol for 10 min, washed carefully with PBS, and blocked with 1% bovine serum albumin at room temperature for more than 1 h. Cultures were then stained with Sporo-Glo (Waterborne Environmental Inc., VA, USA) and 10 µM DAPI for 45 min at 37 °C and mounted on glass slides with ProLong™ Glass Antifade Mountant (Invitrogen). The numbers of parasites and host cells were counted randomly in more than 5 fields of view at a fixed location at 600× magnification for coverslips, and in more than 2 fields of view at a fixed location at 200× magnification for 96 well-plates. At least 500 cells were counted in each well, and three wells were considered as technical repeats for each biological replicate. Each pair of WT and MAPK4-KO samples was counted manually and alternately without distinction of method by means of fluorescence microscopy using BZ-X810 (Keyence, Osaka, Japan). One parasitophorous vacuole of *C. parvum* was considered as a single parasite. The percent of the invasion and infection in MAPK4-KO HCT-8 relative to WT HCT-8 cells was calculated for each biological replicate. Parasites at different developmental stages were classified into trophozoites and meronts based on the size and number of nuclei. The percent of meronts to the total number of parasites was calculated for each biological replicate.

### Attachment assay

For the attachment assay, pre-fixed HCT-8 cells were inoculated to avoid parasite–host interactions following the attachment process. A modified protocol according to previous studies was followed^37,39,62^. Briefly, HCT-8 cells were seeded on 96-well plates (5 × 10^4^ cells/well) and incubated overnight. Each culture was pre-fixed before infection in 3.7% formaldehyde in PBS at room temperature for 15 min and washed three times with PBS. Excysted sporozoites of *C. parvum* were inoculated as described above. Each culture plate was maintained for 30 min for attachment at 37 °C under 5% CO_2_. Indicated samples were further incubated for 24 h. After incubation, each culture was washed three times with ice-cold PBS before fixation and immunofluorescence staining described above. The washing process was carefully performed only once by pipetting to wash out un-attached free sporozoites. The percent of the attachment rates in MAPK4-KO HCT-8 cells to WT HCT-8 cells was calculated for each independent experiment.

### Expression of the MAPK4 gene in MAPK4-KO HCT-8 cells

Total RNA was isolated from WT HCT-8 cells using FastGene™ RNA Basic Kit (NIPPON Genetics Co, Ltd., Tokyo, Japan) and reverse transcribed using the Verso cDNA Synthesis Kit (Thermo Scientific, Waltham, MA USA). To construct the MAPK4 expression vector, cDNA fragments were amplified by primer pair Comp_F and Comp_R listed in Table 1, and ligated into the pCR™-Blunt II-TOPO™ vector (Invitrogen). The sequence was confirmed by sequencing analyses using an ABI PRISM Genetic Analyzer 3130xl (PE Applied Biosystems), and the complete fragment of the MAPK4 coding site was ligated into the pHTN HaloTag^®^ CMV-neo vector (Promega Corporation). The complete sequence of MAPK4 coding site cloned from WT HCT-8 cells is shown as Fig. S1. MAPK4-KO HCT-8 cells were transfected with the constructed vector using Lipofectamine^®^ 3000 reagent (Invitrogen) according to the manufacturer's instructions and incubated two nights for further assessment in the immunofluorescence assay and western blotting.

### Western blotting

A total of 1 × 10^6^ cells of WT, MAPK4-KO, or transfected MAPK4-KO HCT-8 cells were lysed in 100 mL of ice-cold lysis buffer (0.5% Nonidet P-40, 150 mM NaCl, 20 mM Tris–HCl, pH 7.5) containing 1/100 concentration of protease inhibitor cocktail (Nacalai Tesque) for 30 min. Proteins in cell lysates were separated by SDS-PAGE using E-R520L e-PAGEL (ATTO, Tokyo, Japan), transferred to polyvinylidene difluoride membranes (Immobilon-P; Millipore), and blocked with 5% dry skim milk powder in TBS-T buffer (Nacalai Tesque) at room temperature for more than 1 h. The membrane was incubated overnight at 4 ℃ with the anti-HaloTag monoclonal antibody (Promega corporation; 1:1000 dilution) or the anti-beta Actin antibody (66009-1-Ig; Proteintech, Rosemont, IL, USA; 1:1000 dilution), washed three times for five minutes each with TBS-T buffer, and incubated in TBS-T buffer with horseradish peroxidase-conjugated goat α-mouse IgG antibody (PIERCE; 1:5000 dilution) for 1 h at room temperature. The membrane was washed three times for five minutes each with TBS-T buffer and reacted with the Pierce Western Blotting Substrate (Thermo Scientific) to generate chemiluminescence. The signal was detected and analyzed by BioRad ChemiDoc Touch imaging system (Bio-Rad Laboratories, Hercules, CA).

### Apo-ONE homogeneous caspase 3/7 activity assay

Caspase 3/7 activity was assayed in the entire cell culture (both infected and noninfected cells) at 24 h post-infection. The Apo-ONE Homogeneous Caspase-3/7 Assay kit (Promega corporation) was used according to the manufacturer’s suggestions. Briefly, HCT-8 cells were plated in 96-well plates (5 × 10^4^ cells/well) and infected with *C. parvum*, as described above. Each well was lysed with lysis buffer containing caspase substrate Z-DEVD-R100 and incubated at room temperature for 30 min. Caspase-3/7 activity was measured using a fluorescence microplate reader (BMG Lab Technologies, Offenburg, Germany) at 485/535 nm excitation and emission wavelengths.

### Statistical analyses

Each data set represents the mean ± SEM from three biological replicates of at least three technical repeats. All statistical analyses were performed using Excel (Microsoft, Redmond, WA, USA) or R (version 4.2.1.)^63^. The statistical significance of differences between two normally distributed samples was analyzed by using an unpaired two-tailed Student's t test, whereas multiple comparisons were performed according to the Tukey–Kramer method and Two-way ANOVA. A *p* value of < 0.05 was considered statistically significant; *, **, and *** represent *p* < 0.05,* p* < 0.01, and *p* < 0.001, respectively.

## Supplementary Information

Supplementary Information.

## Data Availability

The datasets used and/or analyzed during the current study are available from the corresponding author on reasonable request.
