# Supplementary material for: The role of atypical MAP kinase 4 in the host interaction with Cryptosporidium parvum
Source: Sci Rep. 2023 Jan 19;13:1096. doi: 10.1038/s41598-023-28269-w (PMC9852575; doi:10.1038/s41598-023-28269-w)
Supplement: Supplementary file 1 — Supplementary Information. [file 41598_2023_28269_MOESM1_ESM.pdf]

Supplementary Information

The role of atypical MAP kinase 4 in the host interaction with *Cryptosporidium parvum*

Nina Watanabe[1], Hironori Bando[1,2], Fumi Murakoshi[1,3], Riku Sakurai[1], Mohammad Hazzaz Bin Kabir[1], Yasuhiro Fukuda[1], Kentaro Kato[1,*]

[1] Laboratory of Sustainable Animal Environment, Graduate School of Agricultural Science, Tohoku University, 232-3 Yomogida, Naruko-onsen, Osaki, Miyagi, 989-6711, Japan.

[2] Department of Parasitology, Asahikawa Medical University, 2-1-1-1, Midorigaoka-higashi, Asahikawa, Hokkaido, 078-8510, Japan.

[3] Department of Infectious Diseases, Graduate School of Medical Science, Kyoto Prefectural University of Medicine, 465, Kawaramachi-hirokoji, Kamigyo-ku, Kyoto 602-8566, Japan.

* CORRESPONDENCE

Dr. Kentaro Kato, Ph.D., D.V.M.

Laboratory of Sustainable Animal Environment, Graduate School of Agricultural Science, Tohoku University, 232-3 Yomogida, Naruko-onsen, Osaki, Miyagi 989-6711, Japan.

Phone: +81-229-84-7391, Fax: +81-229-84-7391,

E-mail: kentaro.kato.c7@tohoku.ac.jp

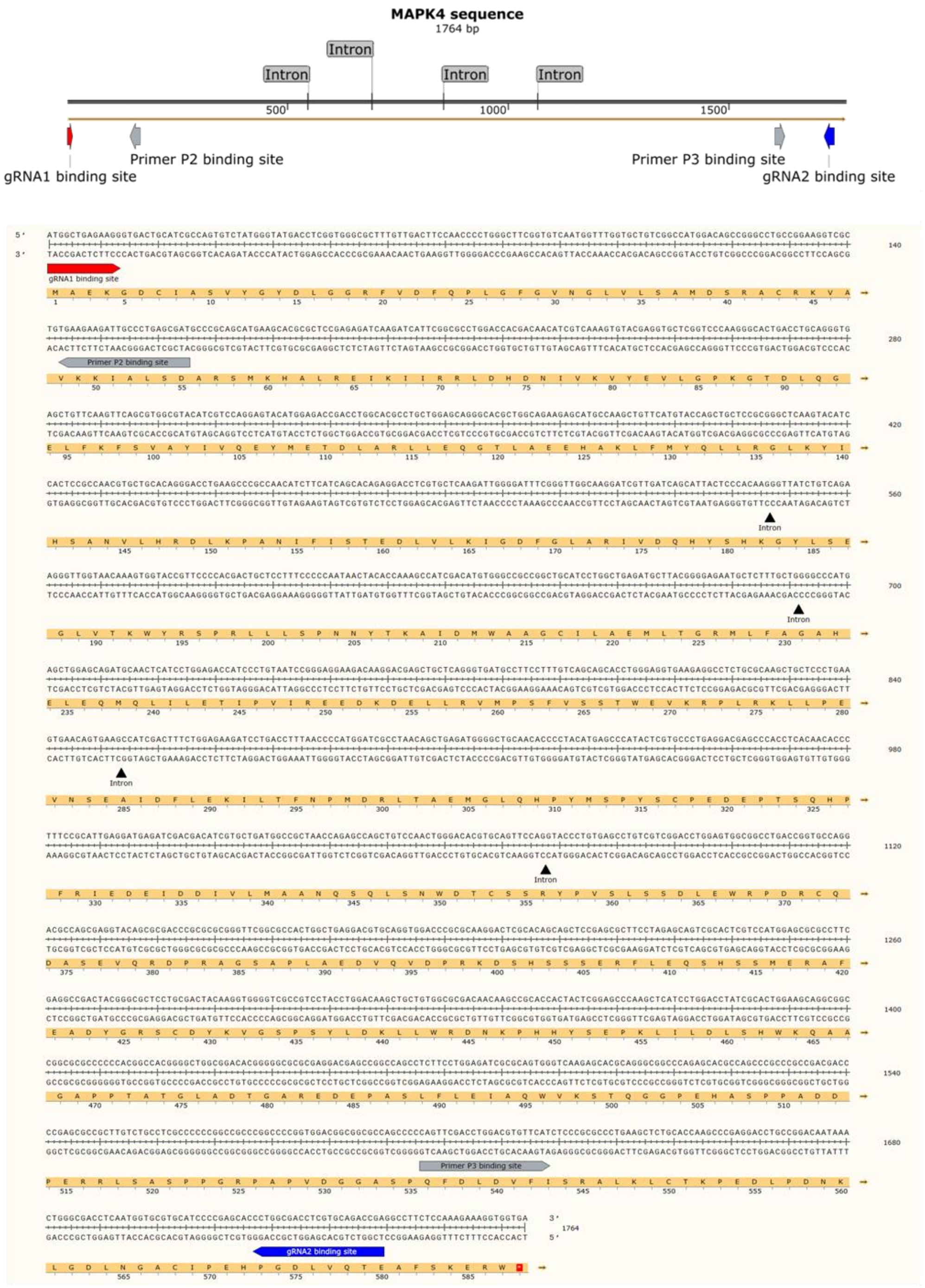

Figure S1. Complete sequence of MAPK4 coding site cloned from WT HCT-8 cells

The images were made using the software Snapgene viewer (version 6.0).

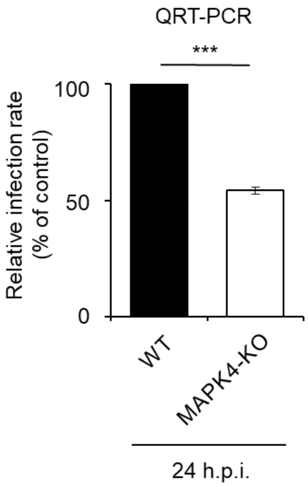

Figure S2. Effect of host MAPK4 deficiency on *C. parvum* infection in HCT-8 cells, confirmed by the QRT-PCR method

Parasite 18S rRNA levels at 24 hours post-infection were detected by QRT-PCR assay and showed similar results to the immunofluorescence assay. The infection rates are shown as the ratio of *C. parvum*-infected amongst total host cells and normalized to the WT HCT-8 cells control. ***, $p< 0.001$ (Student's t test). All graphs show the mean ± SEM for more than three independent experiments.

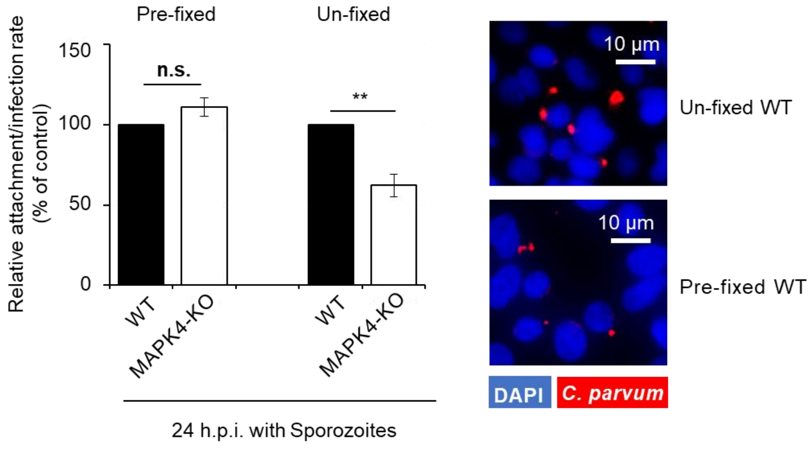

Figure S3. Effect of host MAPK4 deficiency during the attachment stage

Pre-fixed and un-fixed HCT-8 cells were infected with *C. parvum* sporozoites to ensure that excystation processing for sporozoite infection does not affect the parasite viability. The attachment and infection rates were analyzed by use of an immunofluorescence assay 24 hours post-infection. $p = 0.1024$ (Pre-fixed WT - KO) and **, $p < 0.01$ (Student's t test). All images of WT cells are representative of both the WT and MAPK4-KO HCT-8 cells at three independent experiments.

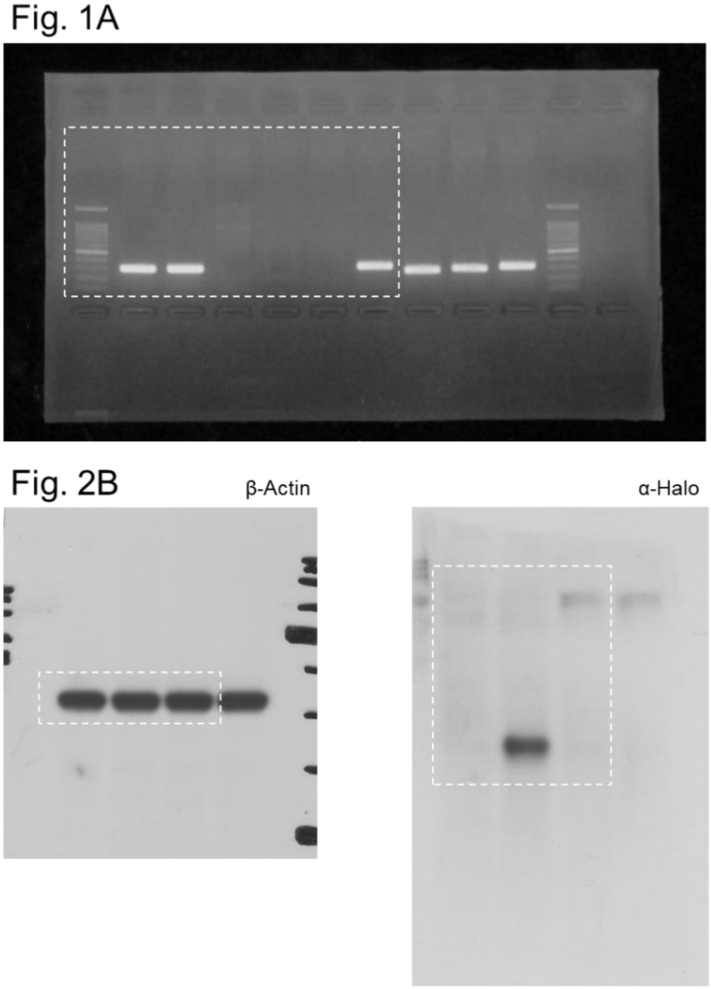

Figure S4. Full Membrane Images for Cropped Data in Figure 1A and 2B

[Supplemental Method]

QRT-PCR Assay for *C. parvum* Detection

HCT-8 cells were seeded 96-well plates (5 × 10^4 cells/well) and infected as described above. A modified QRT-PCR assay was conducted according to previous studies [1,2]. Briefly. Cell monolayers were gently washed 3 times with nuclease-free PBS and total RNA was isolated using ice-cold Bio-Rad iScript qRT-PCR sample preparation reagent (Bio-Rad Laboratories). Parasite growth was assayed using a Qiagen one-step RT-PCR QuantiTect SYBR green RT-PCR kit (Qiagen Inc., Valencia, CA) by detecting parasite 18S rRNA and Human 18S rRNA levels using primers listed in Table 1. All real-time PCR amplification and detection were performed using a Thermal Cycler Dice real-time system (TaKaRa Bio, Shiga, Japan).

[References]

1. Zhang, H., Guo, F. & Zhu, G. Involvement of host cell integrin α2 in *Cryptosporidium parvum* infection. *Infect Immun* **80**, 1753–1758 (2012).
2. Zhang, H. & Zhu, G. Quantitative RT-PCR assay for high-throughput screening (HTS) of drugs against the growth of *Cryptosporidium parvum* in vitro. *Front Microbiol* **6**, 991 (2015).
